# Inconsistencies between Subjective Reports of Cognitive Difficulties and Performance on Cognitive Tests are Associated with Elevated Internalising and Externalising Symptoms in Children with Learning-related Problems

**DOI:** 10.1007/s10802-022-00930-4

**Published:** 2022-07-15

**Authors:** Kira L. Williams, Joni Holmes, Francesca Farina, Maria Vedechkina, The CALM team, Marc P. Bennett

**Affiliations:** 1 Medical Research Council- Cognition and Brain Sciences Unit, University of Cambridge, 15 Chaucer Road, Cambridge, CB2 7EF UK; 2 School of Psychology, University of East Anglia, Norwich, UK; 3 Global Brain Health Institute, Trinity College Dublin, Dublin, Ireland; 4 Present Address: Feinberg School of Medicine, Northwestern University, Chicago, USA

**Keywords:** Functional cognitive difficulties, Working Memory (WM), Inattention, Externalising and internalising difficulties

## Abstract

Children with learning difficulties are commonly assumed to have underlying cognitive deficits by health and educational professionals. However, not all children referred for psycho-educational assessment will be found to have deficits when their abilities are measured by performance on cognitive tasks. The primary aim of this study was to estimate the prevalence of this inconsistent cognitive profile (ICP) in a transdiagnostic sample of children referred by health and education service providers for problems related to attention, learning and memory (*N* = 715). A second aim was to explore whether elevated mental health problems were associated with ICPs. Findings suggest that approximately half of this sample could be characterised as having an ICP. Cognitive difficulties, whether identified by parent ratings or task performance, were associated with elevated internalising and externalising difficulties. Crucially, a larger discrepancy between a parent’s actual ratings of a child’s cognitive difficulties and the ratings that would be predicted based on the child’s performance on cognitive tasks was associated greater internalising and externalising difficulties for measures of working memory, and greater externalising difficulties for measures of attention. These findings suggest that subjective cognitive difficulties occurring in the absence of any task-based performance deficits may be a functional problem arising from mental health problems.

**Supplementary Information:**

The online version contains supplementary material available at 10.1007/s10802-022-00930-4.

## Introduction

Up to 30% of school children receive additional support for learning-related difficulties in the United Kingdom and United States (Department for Education, 2018; National Center for Education Statistics, 2019). Referrals for support typically begin with parent or teacher reports of slow rates of progress in learning, and/or behavioural difficulties such as problems paying attention. In some cases, referrals result in a diagnosis of one or more neurodevelopmental disorders such as attention deficit/hyperactivity disorder (ADHD) and autism following a psychological or psychiatric assessment that draws heavily on subjective reports and observations of a child’s behaviour. Learning difficulties are often linked to deficits in core cognitive domains such as attention and working memory (WM; Follmer, 2018; Holmes et al., 2020; Landerl & Kolle, 2009; Peng & Fuchs, 2016; Peng et al., 2018; Yeniad et al., 2013). However, a common observation in mental health settings is that not every child referred for psycho-educational assessment (based on subjective teacher or parent reports of learning difficulties) performs poorly on performance-based assessments of cognitive ability (e.g. Astle et al., 2019). This means that subjective reports of difficulties can be inconsistent with performance on task-based measures of cognition. In this study, we refer to this as an inconsistent type of cognitive profile (ICP).

The prevalence and aetiology of an ICP has received little attention in child research, despite the potential implications for increased risk of misdiagnoses and inappropriate recommendations for support and intervention. This study sought to estimate the prevalence and symptom profiles of ICPs in a transdiagnostic sample of children and adolescents. Children were first referred to the study by health and education practitioners as experiencing learning-related problems. Their parents then rated their learning, cognitive and mental health difficulties on behaviour rating scales used widely in clinical and educational settings. The children also completed a set of performance-based cognitive tasks. Thus, the recruitment strategy closely resembled the typical routes young people follow for access to educational support and mental health services in the UK.

There is limited information on the prevalence of ICPs among children with learning-related difficulties, but research into the cognitive profiles of individuals with ADHD symptoms provides some insight. ADHD is characterised by elevated levels of inattention and problems inhibiting impulsive and hyperactive behaviours (American Psychiatric Association, 2013). These symptoms are associated with deficits in higher-level cognitive functions such as WM (Castellanos et al., 2006; Lui & Tannock, 2007; Rogers et al., 2011) and behavioural inhibition (Barkley, 1997; Castellanos et al., 2006). Despite this association, studies show that a substantial proportion of children diagnosed with ADHD perform within the typical range on performance-based tests of cognition (Nigg et al., 2005; Willcutt et al., 2005). For example, Solanto et al. (2001) reported that performance-based measures of behavioural impulsivity correctly discriminated 61% of children with ADHD, and performance-based measures of reward sensitivity correctly discriminated 72% of cases. A combination of both captured 88% of cases. Similarly, Nigg et al. (2005) found that 35–50% of children with an ADHD diagnosis had deficits based on performance-based measures of inhibitory cognitive control. The remaining 50–65% were purported to have some alternative aetiology. This is not to say that individuals with ADHD do not have executive function or other cognitive problems. Rather, these findings suggest that many cases of ADHD are not characterised by impaired performance-based assessments of cognition, despite verbal reports of behavioural symptoms that are consistent with poor cognitive control.

A disconnect between verbal symptom reports and biobehavioural markers of cognitive performance is widely documented in later life. A recent systematic review found that approximately 24% of older adults presenting to memory clinics with self-reported memory complaints have age-typical cognitive performance on neuropsychological assessments (McWhirter et al., 2019). Such individuals are described as having functional cognitive difficulties; that is, cognitive difficulties unrelated to brain disease that are secondary consequences of dysregulated attention, meta-cognitive errors and heightened psychological and emotional distress (Hill et al., 2016; Buckley et al., 2013; Farina et al., 2020; McWhirter et al., 2019). In fact, subjective reports of cognitive difficulties are often associated with increased anxiety (Jenkins et al., 2019), depression (Fischer et al., 2008; Schweizer et al., 2018), low self-esteem and somatic complaints (Collins & Abeles, 1996; Hänninen et al., 1994). Performance-based cognitive tasks capture the processing efficiency of cognitive abilities in tightly controlled experimental and structured conditions, but they have been criticised for lacking ecological validity in relation to the day-to-day adaptive use of cognitive skills (Castellanos et al., 2006; Isquith et al., 2013). In contrast, subjective reports or ratings of cognitive problems are open to rater bias (e.g., parental bias; Reid et al., 1987; Stone et al., 2013), but provide a useful measure of functional impairments in cognition (Isquith et al., 2013). Thus, in the case of older adults, a range of emotional difficulties might impact on self-reported functional memory complaints and contribute to their ICP.

We propose the same might be true for some children with learning-related problems. For example, recent studies point to the existence of a discrete subgroup of children presenting with neurodevelopmental difficulties that are derived from emotional rather than cognitive mechanisms (e.g., Karalunas et al., 2019; Nigg et al., 2020a, b, c). For example, Vaidya et al. (2020) identified a subgroup of children with lower emotional regulation and flexibility, in addition to subgroups with lower inhibitory control and other cognitive impairments, in a sample comprised of autistic children, children with ADHD, and those with no diagnosis. Of those with an ADHD diagnosis, 19% had primary problems with emotional regulation and flexibility. Consistent with this, including emotional regulation measures alongside cognitive tasks increases the likelihood of predicting whether a child has neurodevelopmental difficulties: in the case of ADHD, as many as 90% of ADHD cases can be predicted (Sjöwall et al., 2013). Together these studies suggest that emotional and behavioural regulation difficulties likely contribute to the expression of what may appear to be cognitive difficulties in children with neurodevelopmental disorders. However, it has not yet been established whether emotional and behavioural dysregulation are associated with discrepancies between subjective reports and task-based measures of cognition in children referred for learning-related problems. Understanding whether their cognitive abilities reflect mental health difficulties has important implications for clinical services: for some children presenting with cognitive difficulties, therapeutic approaches targeting mental health might be more suitable than educational or cognitive interventions.

The first aim of the current study was to estimate the percentage of children with an ICP among a large transdiagnostic sample identified as experiencing cognitive problems by education and health professionals. It included children with relatively mild problems judged to be compromising their academic progress, who would likely not meet diagnostic thresholds, in addition to many children whose more marked problems would. Some children had a single diagnosis, others had multiple diagnoses, but the majority were undiagnosed despite coming to the attention of a professional for experiencing cognitive difficulties that were affecting their school progress. By adopting a transdiagnostic perspective we were therefore able to include children who were viewed to be experiencing cognitive difficulties by a practitioner, and test whether their cognitive difficulties manifested in both parent’s subjective ratings and on performance-based tasks. To pre-empt the findings, there were some children who did not have difficulties on either the performance-based cognitive tasks or the parent ratings. While they were still viewed as struggling learners by practitioners, we used this group as a comparison sample for those who showed cognitive impairments on our measures.

The cognitive domains we assessed included working memory and attention. These were chosen because they are both implicated in neurodevelopmental disorders and associated with children’s learning outcomes (e.g. Holmes et al., 2014, 2020; Lui & Tannock, 2007; Rubia, 2018). For example, children with working memory problems typically perform relatively poorly on school-based evaluations of learning and standardised measures of reading and maths (e.g., Alloway & Alloway, 2010; Swanson & Sachse-Lee, 2001). Working memory problems are also common in children with a wide range of neurodevelopmental disorders including ADHD (Holmes et al., 2014; Martinussen et al., 2005) and dyslexia (Holmes, 2012; Jeffries & Everatt, 2004). Similarly, children with elevated levels of inattention have impaired reading and maths abilities (e.g., Loe & Feldman, 2007). Attentional difficulties are common among children with neurodevelopmental disorders, most notably those with ADHD (e.g., American Psychiatric Association, 2013).

The two constructs of working memory and attention were treated separately in the analyses. Although they are highly related (e.g., Oberauer, 2019), we treat them as separate constructs, consistent with an extensive literature suggesting that functioning in the two domains make independent contributions to clinical and academic problems (Brocki et al., 2008; Harmer et al., 2002; Holmes et al., 2020; Slattery et al., 2022). To identify children with consistent and inconsistent cognitive profiles we first identified children with subjective cognitive difficulties as rated by their parents. We then split this group into those who had corresponding difficulties on the performance-based tasks (Consistent Cognitive Profile; CCP) and those who did not have impairments on the performance-based tasks (Inconsistent Cognitive Profile; ICP). The cut-offs used to identify whether a child had difficulties was guided by previously established thresholds for high/low cognitive performance (Bondi et al., 2014; Jessen et al., 2020). The definition of an ICP included those with subjective parent ratings of cognitive problems in the absence of difficulties on performance-based tasks, and not the reverse: we did not include those with task-based deficits in the absence of subjectively rated cognitive problems. This due to our interest in identifying whether functional cognitive difficulties – those that are observed in everyday situations and not in tightly controlled experimental settings– are related to mental health problems. This mirrors the work conducted with older adults that has shown people presenting with complaints of everyday memory problems do not always perform poorly on cognitive tasks, but often experience psychological distress (e.g., McWhirter et al., 2019).

A second aim was to investigate whether symptoms of poor mental health were associated with a discrepancy between subjective parental reports of cognitive difficulties and task-based performance. Based on findings from older adults and children with ADHD, we hypothesised that elevated symptoms of mental health difficulties would predict functional cognitive difficulties as captured by parent ratings, even in the absence of impaired cognitive task-performance. The Strengths and Difficulties Questionnaire (Goodman, 1997), a scale used to capture externalising and internalising problems, was used as a measure of mental health. We adopted two statistical approaches to explore whether discrepancies between subjective and performance-based cognitive difficulties were associated with increased mental health problems. First, the effect of cognitive profile (consistent vs inconsistent) on internalising and externalising sub-scales from the Strengths and Difficulties Questionnaire (SDQ; Goodman et al., 2010) was tested. Second, a continuous analysis was conducted to overcome the limitations of using cut-offs to arbitrarily define whether a child had difficulties. The discrepancy between parent ratings and performance-based measures of cognition was quantified using a regression-based approach: residuals were derived that captured the variance in subjective reports of cognitive difficulties not accounted for by performance-based tasks. These values were correlated with the internalising and externalising measures of the SDQ. We expected that both analyses would reveal an association between internalising and externalising difficulties and an ICP.

## Method

### Participants and Protocol

A sample of *N* = 715 (*M* = 9.58 years, *SD* = 2.36, females. *N* = 225) children with complete data on the measures of interest was drawn from the larger (N = 805) Centre for Attention Learning and Memory (CALM) cohort. The CALM cohort is a heterogeneous sample of children aged 5 to 18 years, all of whom were referred to the study for problems in attention, learning and/or memory by health or educational professionals between February 2014 and January 2019. The current sample included *n* = 431 children without a diagnosis (60%) and *n* = 284 (40%) with at least one diagnosis. This also included: *n* = 449 (63%) referred by a professional working in education (e.g., a special-needs teacher or educational psychologist); *n* = 237 (33%) were by a health practitioner (e.g. a paediatrician, clinical psychologist or psychiatrist); and *n* = 28 (4%) referred by a Speech and Language Therapist. There was 1 participant who had missing data for referral type. Parents provided written informed consent and children gave verbal assent for participation. Ethical approval was granted by the National Health Service (NHS) Health Research Authority NRES Committee East of England (13/EE/0157). Children completed standardized measures of cognition and learning during a visit to the CALM research clinic, and parents/carers completed subjective questionnaires relating to cognition, mental health and behaviour. The study protocol and measurement details are described in full in the study protocol (see Holmes et al., 2019).

### Measures

#### Parent-reported Cognitive Measures (Subjective Index)

##### Inattention

The inattention subscale of the Conners 3- Parent Rating Scale Short Form (Conners, 2008) measured subjective attentional difficulties. Parents were asked to rate the frequency of attention-related behaviours over the past month. The subscale consists of 10 items (for example, ‘has trouble staying focused on one thing at a time’ and ‘has trouble changing from one activity to another’), rated using a 4-point Likert scale (0 = Never, 1 = Sometimes, 2 = Often and 3 = Always). Raw scores were converted to T-Scores using age and gender corrected psychometric norms that are described in the Conners user-manual (Conners, 2008).

##### WM

The WM subscale of the Behavioural Rating Inventory of Executive Function (BRIEF) measured subjective WM difficulties (Gioia et al., 2000). Here, parents were asked to rate the frequency of 10 WM-related behaviours over the past 6 months (e.g. ‘when given three things to do, remembers only the first or last’). Each item was rated for frequency over the past six months using a Likert Scale (0 = Never, 1 = Sometimes, 2 = Often and 3 = Always). Raw scores were standardized into T-Scores as per the BRIEF user-manual (Gioia et al., 2000).

#### Performance-based Cognitive Measures (Objective Index)

##### Attention

The Barking/Vigil (B/V) sustained attention subtest of the Test of Everyday Attention in Children 2 (TEA-Ch2; Manly et al., 2016) provided an objective performance-based measure of attention. Participants counted the number of auditory items (barks for children aged under 8 years, bleeps for those aged 8+) heard at random intervals over the course of ten trials. A score was awarded for the number of correct trials ranging from 0 to 10. Raw scores were converted by the software to scaled scores derived from population means (see supplementary materials, Table S1).

##### WM

The Backward Digit Recall (BDR) subtest of the Automated Working Memory Assessment (AWMA; Alloway, 2007) provided an objective performance-based measure of WM. BDR involves immediate serial recall of sequences of spoken digits in reverse order. It is a span task with six trials at each span length, with the maximum list length set to seven digits. The task automatically progresses up a span level, adding a digit to the list if there are four or more correct answers out of the six trials at any span length. The test is discontinued following three or more incorrect responses at any span length. A score was awarded for the number of correct trials. Raw scores were converted by the software to standard scores derived from population means (see supplementary materials, Table S1).

#### Mental Health

The Strengths and Difficulties Questionnaire (SDQ) comprises 25 items that capture emotional symptoms, conduct problems, hyperactivity, peer problems and prosocial behaviour (Goodman, 1997). Parents rated their child’s behaviour over the past six months for each subscale using a Likert scale (0 = Not True, 1 = Somewhat True and 2 = Certainly True). The externalising subscale of the SDQ is calculated by combining the conduct problems and hyperactivity scales. The internalising subscale is calculated by combining the emotional problems and peer problems scales (Goodman et al., 2010). We chose to use the combined internalising and externalising scores to capture the most common and broad dimensions of mental health (e.g., Achenbach & Edelbrock, 1981) and because of the controversy over the differentiation between what is measured between the five subscales (e.g. Goodman et al., 2010). All raw scores were converted into T-scores using the British norms presented in Meltzer et al. (2000).

### Data Coding and Analysis

The parent-reported attention/WM scales were recoded to be in the same direction as the performance-based tasks such that lower values tend towards cognitive difficulties. Standard scores were converted to Z-scores using the population means and SDs. This ensured all tasks were on the same scale but enabled us to retain the population-level values for establishing whether children had cognitive difficulties. That is, the Z-scores used to determine cognitive profiles were based on population, and not sample means. Two analytic approaches were adopted to explore the mental health symptoms associated with ICPs. These are described below.

#### Categorical Approach

**Fig. 1:**
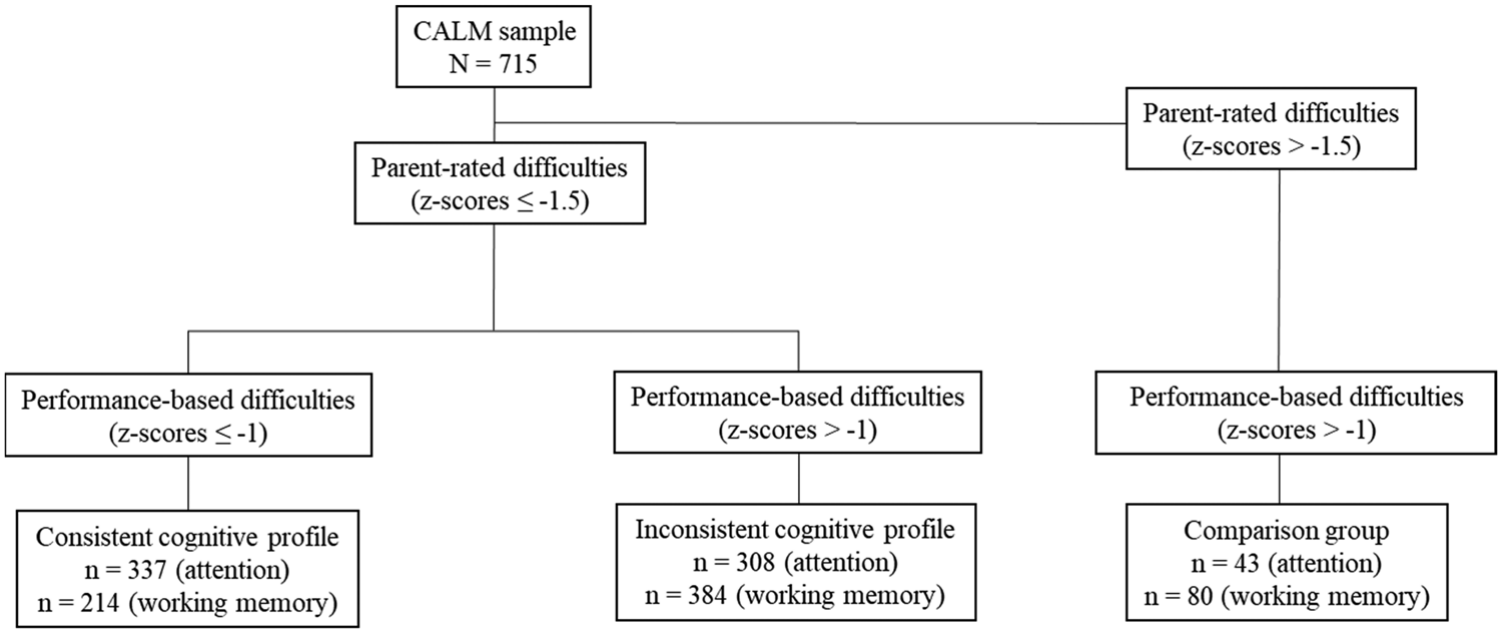
Schematic overview of the steps and thresholds used to group participants as having a consistent cognitive profile (CCP) and inconsistent cognitive profile (ICP) and to determine the comparison group who did not meet the threshold for neither parent-reported or performance-based cognitive difficulties. These steps were taken for both attention and working memory separately, and final group numbers are reported

Three groups were identified and considered in the analysis: children with ICPs, those with CCPs, and a comparison group who were rated as having age-typical cognitive abilities and performed in the age-typical range on the cognitive tasks (see Tables 1 and 2; Supplemental Materials, Table S2). The procedure for categorising participants as having ICPs or CCPs is displayed in Fig. 1. To summarise, it was conducted in two stages. First, children were classified as having / not having subjective cognitive difficulties as rated by their parents: only those with parent-rated subjective cognitive difficulties were selected. Second, those with parent-rated subjective cognitive difficulties were split into those who had corresponding difficulties on performance-based tasks (CCP) and those who did not have impairments on the performance-based tasks (ICP). This grouping was conducted separately for each cognitive domain (i.e., groupings based only on the attention/inattention measures and grouping based only on the WM measures).

**Table 1:** Profiles of cognitive difficulties

*Profile label*	Parent-rated difficulties	Performance-based difficulties
Inconsistent	✔	✘
Consistent	✔	✔
Comparison group^a^	✘	✘
Performance-based difficulties only^b^	✘	✔

Two thresholds to operationalise ‘cognitive difficulties’, according to parent-ratings and performance-based ratings, were explored in the current study. These were 1 SD and 1.5 SD below the population mean. Psychometric norms for performance-based measures are provided in supplemental materials

^a^Children in this group did not pass the thresholds for impaired cognition using parent-reports and performance-based tasks. They were however referred at recruitment because of some learning-related difficulty. This groups is therefore likely to represent children with elevated but sub-threshold cognitive difficulties

^b^Children in this group did not pass the threshold for impaired cognition using parent-reports. Given their scarcity (fewer than 5% of the CALM sample), these data are not presented

There is no consensus on a precise cut-off to define impaired cognitive performance. A cut-off value of 1 SD below the population mean has been recommended when determining cognitive difficulties using two independent performance-based measurements, while a more stringent cut-off value of 1.5 SD below the population mean is commonly used when a single measure is used (Bondi et al., 2014; Jessen et al., 2020). These boundaries are inherently arbitrary but are simply intended to provide useful thresholds for research purposes (Albert et al., 2011). As this was an exploratory study, cut-off values of both 1.5 and 1 SD below the population mean were used to identify participants with ICPs.

Participants were classified separately using both liberal (Z-scores ≤ -1) and conservative (Z-scores ≤ -1.5) cut-offs for subjective parent ratings in each cognitive domain. In anticipation of the results section, the placement of this threshold had a negligible impact on prevalence estimates. The final analysis was therefore based on the conservative definition of subjective cognitive difficulties (Z-scores ≤ -1.5), meaning participants with a Z-score > -1.5 on subjective ratings were not included.

Those who were retained (Z-scores ≤ -1.5 on the subjective rating scales) were next categorised into ICPs and CCPs based on their scores on the performance-based tasks of attention and WM. Again, conservative and liberal cut-offs were used to define the children’s difficulties. Using the conservative cut-off, a CCP was defined by Z-scores ≤ -1.5 on the performance-based tasks. These children therefore had cognitive difficulties as reported by their parents and on the task-based measures. The remaining participants with a Z-score > -1.5 on the performance-based tasks were classified as having an ICP (i.e. cognitive difficulties reported by parents but age-typical performance on the task-based measures). Using the liberal cut-off, participants with a Z-score ≤ -1 on the performance-based tasks were classified as having a CCP and the remaining participants with a Z-score > -1 on the tasks were classified as having an ICP. In anticipation of the results section, the final analysis reports the liberal definition of objective cognitive difficulties. This is because it demonstrated better sensitivity and specificity for distinguishing cognitive profiles.

Children with age-typical cognitive performance, both rated by their parents and on the performance-based task measures, were used as a comparison group. The prevalence of children in the sample with an ICP and a CCP was calculated based on the percentage of children with each profile relative to the whole sample for whom we had complete data (*N* = 715). ANOVAs were used to compare internalising and externalising symptoms between the ICP, CCP and comparison groups.

#### Continuous Approach

**Fig. 2:**
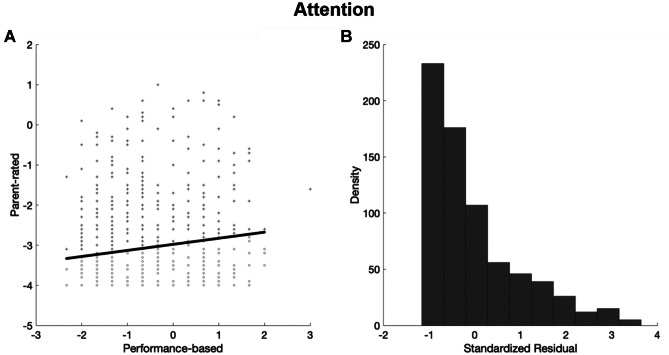
Residual plots for the continuous analysis for measures of attention. Residuals represent the difference between the actual parent-rated difficulties for each child and the parent-rated difficulties predicted by actual performance on a sustained attention task. (**A**) This figure indicates the regression line; values above this line (relatively more positive) indicate cases wherein parent report less severe inattention than would be predicted by performance. Values below this line (relatively more negative) indicate cases wherein parents report more severe inattention than would be predicted by performance. (**B**) The distribution of residual scores

**Fig. 3:**
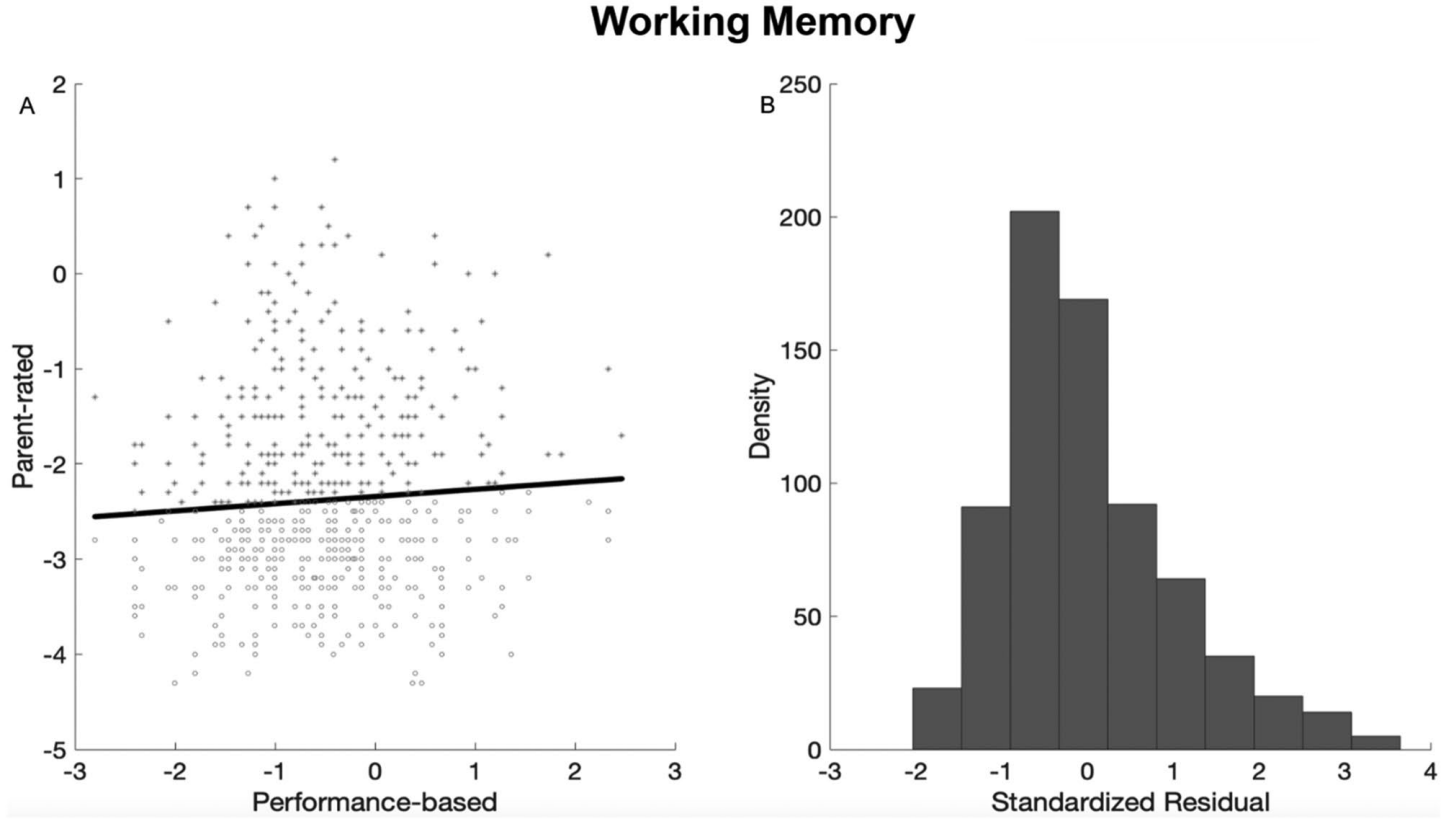
Residual plots for the continuous analysis for measures of working memory. Residuals represent the difference between the actual parent-rated difficulties for each child and the parent-rated difficulties predicted by actual performance on a working memory task. (**A**) This figure indicates the regression line; values above this line (relatively more positive) indicate cases wherein parent report fewer WM difficulties than would be predicted by performance. Values below this line (relatively more negative) indicate cases wherein parents report more WM difficulties than would be predicted by performance. (**B**) The distribution of residual scores

For every participant with parent-rated cognitive difficulties (Z-scores ≤ -1.5 on the subjective rating scales), a residual was created to estimate discrepancies between parent ratings of cognitive difficulties and task-based cognitive performance. To do this, task-based performance was regressed onto subjective parent-ratings and the standardised residual scores were saved for each individual. The residuals proxied the difference between each child’s parent-rated difficulties and the level of parent-rated difficulties predicted by an individual’s performance (Figs. 2 and 3). Positive residuals indicated that parent-ratings were lower than the child’s task-based performance would predict (e.g., the child was rated as having fewer difficulties than their task-based performance would predict). Negative residuals indicated that child’s parent-rated difficulties were higher (e.g., more severe), than predicted by their task-based performance. Children with negative residuals were selected for inclusion in the subsequent continuous analysis. This was because of our interest in cases where subjective ratings of cognitive difficulties were heightened relative to a child’s task-based cognitive performance. In other words, we captured the profile most aligned with the study aims and the approach used in the categorical analysis.

Negative residuals were transformed such that lower values indexed when task-based performance accurately predicted subjective parent-ratings. Higher values therefore indexed cases where task-based performance less accurately predicted subjective parent ratings. In other words, the larger the value, the larger the discrepancy between objective cognitive task performance and parent ratings of cognitive difficulties: this was always in the direction that parent rated difficulties were more severe than expected based on the child’s performance on a cognitive task. From here on, these values are referred to as inconsistency coefficients. We explored the association between these values, demographic information, and measures of internalising and externalising difficulties using regression models.

## Results

### Category-based Analysis

The number of children with CCPs and ICPs based on the different cut-offs are presented in Tables 2 and 3. Shifting the threshold for parent-rated cognitive difficulties from 1 SD (Table 2) to 1.5 SD (Table 3) below the population mean resulted in a small change in prevalence (mean ± *SD* change in prevalence = 2.68 ± 3%). The primary analyses therefore focused on the more conservative definition of subjective cognitive difficulties (1.5 SD below the population mean (Z ≤ -1.5) seen in Table 3.

**Table 2:** Cognitive profile prevalence using an inclusion threshold of 1 SD below the population norms for parent-ratings of cognitive difficulties

	**Attention**				**Working Memory**			
	*Performance-based task threshold*							
*Profile labels*	1 SD below mean		1.5 SD below mean		1 SD below mean		1.5 SD below mean	
	**n**	**%**	**n**	**%**	**n**	**%**	**n**	**%**
Inconsistent	318	44	463	65	420	59	582	81
Consistent	346	48	201	28	230	32	68	10
Comparison*	33	5	44	6	44	6	63	9

Prevalence is expressed as a percentage of the overall CALM sample with full data (N = 715)

Standard deviations are based on population norms for each performance-based task

^*^Children in this group did not pass the thresholds for impaired cognition using both parent-reports and performance-based measures

**Table 3:** Cognitive profile prevalence using an inclusion threshold of 1.5 SD below the population norms for parent-ratings of cognitive difficulties

	**Attention**				**Working Memory**			
	*Performance-based task threshold*							
*Profile labels*	1 SD below mean		1.5 SD below mean		1 SD below mean		1.5 SD below mean	
	**n**	**%**	**n**	**%**	**n**	**%**	**n**	**%**
Inconsistent	**308**	43	448	63	**384**	54	533	75
Consistent	**337**	47	197	28	**214**	30	65	9
Comparison*	**43**	6	59	8	**80**	11	112	16

Prevalence is expressed as a percentage of the overall CALM sample with full data (N = 715)

Standard deviations are based on population norms for each performance-based task

Group numbers highlighted in bold represent the final groups used to compare internalising and externalising difficulties

^*^Children in this group did not pass the thresholds for impaired cognition using both parent-reports and performance-based measures

Increasing the threshold for performance-based cognitive difficulties from 1 SD to 1.5 SD below the population mean resulted in a larger change (mean ± *SD* change in prevalence = 4.81 ± 17%). Almost all children were classified as having an ICP using the more conservative cut-off value of 1.5 SD below the population mean. For this reason, the primary analyses reported here focus on the more liberal definition of performance-based cognitive difficulties (1 SD below the population mean; Z ≤ -1), as it demonstrated better sensitivity and specificity for distinguishing cognitive profiles.

For clarity, all children included in the primary analyses had subjective parent-rated cognitive difficulties 1.5 SD below the population mean. Within this, those with performance-based cognitive scores at least 1 SD below the population mean were classified as having a CCP, and those with performance-based cognitive scores greater than 1 SD below the population mean were classified as having an ICP (see Table 1 for a summary). Descriptive statistics are displayed in Table 4.

**Table 4:**
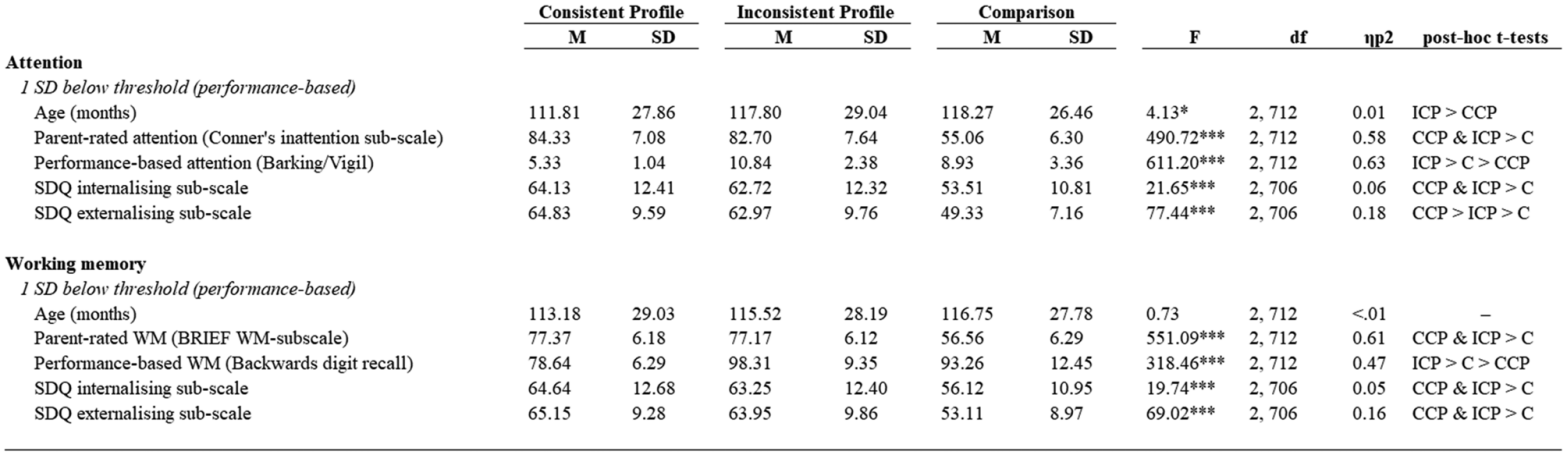
Summary of profile means, standard deviations and main effects

Participants included in the consistent cognitive profile (CCP) and inconsistent cognitive profile (ICP) were 1.5 SD below the population mean on parent-reported difficulties

The comparison group did not pass the threshold for parent-rated nor performance-based difficulties but were referred on the basis of learning-related difficulties

*p < 0.05, **p < 0.01, *** p < 0.001

#### Attentional Difficulties

The prevalence of a CCP was 47% (n = 337, 111 female), while 43% (n = 308, 83 female) of participants evinced an ICP (Table 3). The ICP group was characterised by heightened subjective parent ratings of inattention but no performance impairments on a sustained attention task. Finally, 6% (n = 43, 21 female) of participants formed a comparison group, with scores in the age-typical range on both the parent ratings of inattention and performance on the sustained attention task.

The ICP group were significantly older than the CCP group (t (df) = 2.69 (643), p < 0.008, d = 0.21) but did not differ in terms of sex (χ^2^ (df) = 2.75 (1), p = 0.098). A three-way ANOVA revealed a main effect of group (CCP vs. ICP vs. comparison group) on externalising symptoms (F(2,680) = 49.82, p < 0.001, partial eta^2^ = 0.13). Post-hoc t-tests indicated that the comparison group showed fewer externalising symptoms than both the CCP (t (df) = 9.98 (374), p < 0.001, d = 1.66) and ICP groups (t (df) = 8.73 (348), p < 0.001, d = 1.44). In addition, the ICP group demonstrated fewer externalising symptoms than the CCP group, after using a Bonferroni corrected alpha level (t (df) = 2.47 (638), p < 0.04, d = 0.19).

A three-way ANOVA revealed a main effect of group on internalising symptoms (F(2,680) = 11.88, p < 0.001, partial eta^2^ = 0.03). Post-hoc t-tests indicated that the comparison group showed fewer internalising symptoms than both the CCP (t (df) = 4.86 (374), p < 0.001, d = 0.79) and ICP groups (t (df) = 4.13 (348), p < 0.001, d = 0.68). However, there was no difference between the ICP and CCP groups (t (df) = 1.46 (638), p = 0.436). Descriptive statistics are displayed in Table 4. This suggests that both children with CCPs and ICPs have greater externalising and internalising difficulties than children with no parent-rated or performance-based cognitive difficulties, and that children with CCPs have greater externalising difficulties than those with an ICP.

#### Working Memory Difficulties

The prevalence of participants with a CCP was 30% (n = 214, 77 female), and 54% (n = 384, 110 female) had an ICP (Table 3). The ICP group was characterised by elevated subjective parent-ratings of WM difficulties but no impairments in WM task performance. Finally, 11% (n = 80, 26 female) of participants had age-typical working memory abilities, as measured by both parent ratings and performance on the WM task.

There were no significant differences in age (t (df) = -0.97 (596), p = 0.335) or sex across the groups. (χ^2^ (df) = 3.44 (1), p = 0.06). A three-way ANOVA revealed a main effect of group (CCP vs. ICP vs. comparison group) on externalising symptoms (F(2,669) = 45.75, p < 0.001, partial eta^2^ = 0.12). Post-hoc t-tests indicated that the comparison group showed fewer externalising symptoms than both the CCP (t = 9.19 (288), p < 0.001, d = 1.25) and ICP groups (t (df) = 8.80 (460), p < 0.001, d = 1.065). However, there was no difference between the ICP and CCP groups (t (df) = 1.46 (590), p = 0.433).

A three-way ANOVA revealed a main effect of group on internalising symptoms (F(2,669) = 12.01, p < 0.001, partial eta^2^ = 0.04). Post-hoc t-tests indicated that the comparison group showed fewer internalising symptoms than both the CCP (t (df) = 4.84 (288), p < 0.001, d = 0.64) and ICP groups (t (df) = 4.25 (460), p < 0.001, d = 0.53). However, there was no difference between the ICP and CCP groups (t (df) = 1.31 (590), p = 0.568). Descriptive statistics are displayed in Table 4. This suggests that both children with CCPs and ICPs have elevated internalising and externalising symptoms compared to children with no parent-rated or performance-based WM difficulties. However, there is no significant difference in mental health symptoms between children with an ICP or CCP for WM.

### Continuous Analysis

Correlations between all the measures are provided in the Supplemental Materials (Fig. S1). The parent rated measures of inattention and working memory were significantly associated with each other, but still had a moderate proportion of variance not explained by the other. Both were significantly associated with externalising and internalising symptoms, but with a weaker association to internalising symptoms. The performance-based measures of attention and working memory were significantly associated with one another, but with a weak correlation. Despite being significantly associated, attention tasks were only weakly related to the parent ratings of cognition and externalising symptoms, whilst working memory tasks were only significantly associated with parent rated and performance based attention tasks, but with a weak correlation.

#### Attentional Difficulties

An inconsistency coefficient was derived by regressing sustained attention task performance on to the inattention ratings and saving the negative value residuals (see Fig. 2 and description of this method in the Analysis Plan). The residual plot presented in Fig. 2 shows the discrepancy was skewed towards parent-rated inattention ratings that were more severe than those predicted by performance on the sustained attention task.

The inconsistency coefficient was significantly associated with externalising symptoms, *Adj* R^2^ = 0.01, *p* = 0.007. This means that parent-rated inattention was poorly predicted by sustained attention task performance in children with heightened levels of externalising problems, *ß* = 0.06, *p* = 0.007. It was not associated with internalising symptoms, *Adj* R^2^ < 0.01, *p* = 0.09.

The inconsistency coefficient was not related to age, *Adj* R^2.^ = 0.01, p = 0.078, but was greater for girls (M = 0.66, SD = 0.25; n = 142) than for males (M = 0.60, SD = 0.29; n = 311). This means that parent ratings of inattention were more poorly predicted by task performance for girls than boys.

#### Working Memory Difficulties

An inconsistency coefficient was derived by regressing backwards digit recall task performance on to parent-rated working memory difficulties and saving the residuals (see Fig. 3, and description in the Analysis Plan). The residual plot presented in Fig. 3 shows the discrepancy was skewed towards parent-rated WM difficulties that were more severe than those predicted by performance on the WM task.

The inconsistency coefficient was significantly associated with externalising symptoms, *Adj* R^2^ = 0.04, *p* < 0.001, and internalising symptoms. *Adj* R^2^ = 0.03, *p* < 0.001. Specifically, parent-rated inattention was poorly predicted by cognitive task performance in children with heightened levels of externalising, *ß* = 0.14, *p* < 0.001, and internalizing difficulties, *ß* = 0.10, *p* < 0.001.

The inconsistency coefficient for WM was associated with age, *Adj* R^2.^ = 0.03, p < 0.001. Parent-rated WM difficulties were poorly predicted by WM task performance in older children, *ß* = 0.002, *p* < 0.001. The inconsistency coefficient was greater for girls (M = 0.83, SD = 0.45; n = 135) than boys (M = 0.58, SD = 0.40; n = 284), suggesting that parent ratings of WM problems were more poorly predicted by WM task performance in girls than boys.

## Discussion

Many children with learning-related problems have associated cognitive difficulties. However, not every child referred for psycho-educational/clinical assessment based on a practitioner’s observation of learning problems performs poorly on performance-based measures of cognitive function. The current study estimated the prevalence of this inconsistent cognitive profile (ICP) in a large sample of children referred for problems in attention, learning and / or memory by health and education practitioners, and explored whether inconsistencies between subjective ratings and performance-based tests of cognitive problems were associated with elevated internalising and externalising symptoms. ICPs were highly prevalent in the sample. Children with ICPs and those with consistent cognitive profiles (CCPs; both parent-rated cognitive difficulties and impaired cognitive task-performance) had elevated levels of internalising and externalising problems relative to children with age-typical cognition. Children classified as having CCPs for attention had greater externalising problems than those with ICPs, but there were no other differences between these two groups. Discrepancies between the ratings of working memory difficulties provided by parents and those predicted by performance on tasks of working memory were associated with increased symptoms of internalising and externalising difficulties. For measures of attention, these discrepancies were only associated with externalising difficulties. These findings are discussed in turn below.

Our unique transdiagnostic sample of over 700 children was comprised entirely of individuals who were identified by health or educational providers as experiencing cognitive or learning problems that were affecting their school progress. Among these, 47% had an ICP for attention and 54% for WM: in both cases, almost half of the sample had an inconsistency between subjective parent-ratings of cognitive difficulties and their performance on task-based measures of cognition. More children had a consistent pattern of difficulties across subjective parent ratings and performance-based measures for attention than WM; 43% compared to 30%. These differences might reflect the ease with which attentional lapses and difficulties sustaining attention can be observed, and the relative familiarity parents will have with attention-based problems. It is comparatively easy to observe when a child loses focus or becomes distracted. In contrast, identifying working memory failures is less commonplace, and the concept of working memory is less well integrated into everyday language. Differences in parents understanding of attentional and working memory failures might therefore underlie the differences in consistencies between their ratings and task-based performance across the two cognitive domains.

Our findings suggest that almost half (43–54%) of children with practitioner-observed cognitive and learning-difficulties do not exhibit any deficit on performance-based measures of WM and sustained attention, which contrasts the reported ubiquity of these cognitive difficulties in struggling learners (Follmer, 2018; Holmes et al., 2020; Landerl & Kolle, 2009; Peng & Fuchs, 2016; Peng et al., 2018; Yeniad et al., 2013). This figure is consistent with the percentage of unclassified children from ADHD discriminatory studies (Nigg et al., 2005; Solanto et al., 2001), and studies exploring functional cognitive difficulties in older adults (McWhirter et al., 2019; Minett et al., 2008; Hill et al., 2016). These findings highlight the importance of assessing children’s performance on cognitive tasks in addition to observing their behaviours to fully understand where their difficulties lie. This will help determine appropriate approaches to intervention.

Children with parent-reports of cognitive difficulties, either as part of an ICP or CCP, were rated as experiencing greater internalising and externalising difficulties than children who did not meet the threshold for attention and WM difficulties. This is consistent with our hypothesis that children with higher subjective parent ratings of difficulties in attention and WM, even in the absence of performance-based deficits in these areas, would experience more symptoms of internalising and externalising problems. It also aligns with literature indicating that children with neurodevelopmental difficulties are at increased risk of mental health problems (e.g. Bryant et al., 2020; Francis et al., 2019; Holmes et al., 2021), and supports growing links between emotional processes and learning-related difficulties (Nigg et al., 2020a, c; Vaillancourt et al., 2017; Yoshimasu et al., 2012).

While elevated mental health problems were not specific to children with an ICP, we did find an association between an ICP and elevated internalising and externalising symptoms consistent with our hypothesis. Although this study is cross-sectional and will require further research to establish causal pathways, we tentatively propose that everyday cognitive difficulties in these children may arise, in part, through mental health problems as they do for older adults (e.g. McWhirter et al., 2019): negative mood states might impair cognitive functioning. Indeed, the cognitive load associated with down regulation of negative emotional states is greater in younger children than in adults and can lead to an increase in everyday cognitive failures irrespective of baseline ability (Scheibe & Blanchard-Fields, 2009). These cognitive failures might occasion subjective reports of cognitive difficulties from parents and/or educators. Evidence suggests that cognitive failures may also trigger environmental consequences that further impede performance and psycho-social functioning (Sonuga-Barke, 2005; Nigg et al., 2005). First, a sense of failure can negatively impact mood and sense of self, which exacerbates the original negative affective states (Farina et al., 2020). Second, the anticipation of cognitive difficulties is associated with increased cognitive fatigue and performance deficits (Lenaert et al., 2021). Third, contexts associated with cognitive failures may become aversive and prompt withdrawal, thus limiting opportunities for cognitive growth and academic and social development (Sonuga-Barke, 2005; Loe & Feldman, 2007). Such consequences can also increase opportunities for subjective reports of cognitive difficulties from parents and/or educators. In this way, we tentatively propose that subjective reports of cognitive difficulties that occur without any performance-based deficits may be a functional consequence of mental health difficulties. This requires empirical testing via future experimental and longitudinal studies.

Externalising problems were more common among children with CCPs in attention than in children with an ICP in attention. This suggests that children with attentional difficulties measured by both parent ratings and task performance are more hyperactive and experience more conduct problems than children with subjective ratings of attentional problems without problems on a sustained attention task. This was an unexpected finding, which might be explained by theories ascribing a core role to cognitive control in behavioural regulation (e.g. Barkley, 1999; Brocki & Bohlin, 2006; Casey et al., 2001; Scheres et al., 2004). Sustained attention tasks require both focus and the ability to inhibit distractions. Those with a CCP, who performed poorly on the task, may therefore have poorer cognitive control or inhibitory skills than those with an ICP.

The pattern of associations found between discrepancies in subjectively rated cognitive problems and task-based measures of working memory and attention using a categorical grouping approach was largely replicated when a continuous approach to the analysis was adopted. In these analyses, parent rated difficulties were poorly predicted by task performance in children with heightened levels of externalising problems for both working memory and attention. This aligns with the findings from the categorical analysis showing that children with subjective ratings of cognitive difficulties in the absence of task-based deficits had elevated externalising problems relative to the comparison group. Similarly, a larger inconsistency between the parent reports of working memory problems, and those predicted by performance on working memory tasks, was associated with elevated internalising symptoms. This was consistent with the outcomes of the group-based approach showing that children with an ICP for working memory had elevated internalising symptoms relative to the comparison group.

The only inconsistency across the methods was that an ICP for attention was associated with increased internalising symptoms relative to the comparison group in the categorical analysis, yet internalising difficulties were not associated with the variance remaining in parent reported attentional problems after the variance predicted by task-performance had been removed. The continuous analysis is likely more sensitive than the categorical, suggesting subjective everyday problems with attention might not be related to functional problems arising from internalising symptoms. Our data therefore suggest internalising symptoms explain more of the discrepancies between everyday difficulties and task performance for working memory than for attention, and that externalising symptoms may explain some of the discrepancies between everyday difficulties and task performance for both attention and working memory. This pattern is consistent with earlier work reporting associations between working memory and depression, but not between attention and depression (Matthews et al., 2008), and links between working memory and internalising and externalising symptoms (Donati et al., 2021).

A final noteworthy finding is that parent ratings of difficulties in attention and working memory were more poorly predicted by task performance for girls than boys. This suggests parents may have been less able to detect or observe cognitive difficulties in girls. This might reflect implicit gender biases and stereotyping (e.g., discussed in Anderson, 1997; Sciutto et al., 2004) that lead parents to rate boys as experiencing more difficulties. Alternatively, it might be driven by different expressions of difficulties in boys and girls, and in particular by the tendency for girls to mask their problems in everyday situations (e.g., Dhuey & Lipscomb, 2010; Hiller et al., 2014; Hull et al., 2020).

Drawing together the findings from both analytic approaches, cognitive difficulties, whether part of a CCP and an ICP, are associated with increased externalising and internalising problems. Further, when cognitive task performance is a poor predictor of subjective everyday cognitive difficulties, externalising symptoms predict functional impairments in working memory and attention, and internalising symptoms predict functional impairments in working memory. Cognition and mental health interact across development. The cognitive reserve hypothesis suggests poor cognitive function impairs the downregulation of negative emotional responses, such as worry or sadness, leading to poor mental health (LeMoult & Gotlib, 2019). Conversely, the interference hypothesis suggests psychological distress disrupts cognitive processing by shifting cognitive resources away from task-relevant information and onto negative thoughts (Llewellyn et al., 2008; Stawski et al., 2006), resulting in both short- and long-term cognitive difficulties (Dolcos et al., 2020). The dynamic mutualism hypothesis integrates these two opposing theories, arguing that mental health and cognitive function reciprocally interact over time, leading to a dynamic cycle of exacerbation across the lifespan (Fuhrmann et al., 2020). It is not possible to tease apart these hypotheses based on the current data, but our findings do add to a growing body of work demonstrating associations between cognitive function and mental health in childhood.

### Limitations and Future Directions

It was necessary to nominate cut-off criteria to assign children to the ICP and CCP groups. Despite their common use in both research studies and clinical and educational practice, the choice of cut-off values is somewhat arbitrary. For this reason, we used two different cut-off values to establish the prevalence estimates of each profile (1 SD and 1.5 SD below the population mean, as commonly recommended; Jessen et al., 2020). We report the final analyses comparing the ICP and CCP groups using the more conservative -1.5 SD cut-off for the subjective cognitive ratings and the -1 SD for the performance-based tasks. The decision to use different values for the two groupings was guided by the sample characteristics. All children were referred to the study by a practitioner who judged them to be experiencing cognitive and learning problems. This resulted in a bias towards most children being rated as having subjective cognitive difficulties by their parents who knew why the children had been referred (caregiver concern). For this reason, we adopted the more stringent cut-off to define difficulties on the subjective rating scales. To ensure there were sufficient children in the consistent group who had poor performance on cognitive tasks, a more liberal cut-off was adopted. This was because a more stringent cut-off resulted in a small percentage of the sample being identified as having performance-based cognitive difficulties. Given that children were referred for cognitive difficulties, the more liberal cut-off improved sensitivity and specificity for the sample. Thus, because of the nature of our sample, we adopted different criteria across the measures to ensure we had sufficient children in the ICP and CCP groups. Future studies with children with a broader range of scores on multiple tests of subjective and performance-based tests are needed to test the robustness of our findings.

It is possible that the association between subjective reports of cognition and of mental health simply reflect common variance as the ratings were provided by one informant (parent) who may be influenced by caregiver concern. Although we cannot exclude this possibility, the data suggest the subjective ratings provided meaningful measurements of children’s cognitive skills and psychological functioning because they showed different patterns of association for different children. Future studies should include ratings from other informants, including teachers and clinical practitioners.

Research shows that subjective reports and performance-based measures of cognition are not highly correlated, suggesting they may be capturing different abilities, or different aspects of cognitive function (Isquith et al., 2013; Toplak et al., 2017). This might explain why there are inconsistencies between parent ratings and performance-based measures. Again, we cannot rule this out, but there was a strong correspondence between both measurement types in 30–40% of children, which provides us with some confidence in the data.

While our novel sampling approach enabled us to recruit children who were observed to have everyday functional cognitive and learning problems in the absence of performance-based task deficits, which was critical to addressing the study goals, it is unclear whether our findings will generalize to samples recruited using different selection criteria. Future studies exploring functional cognitive difficulties in community samples may also be useful to both introduce greater variance in cognitive function to distinguish small effects between ICPs and CCPs, and to increase the generalisability to the general school-age population. Related to this, due to the sample spanning a wide age range and all being referred for difficulties at school, we used population means from standardised test manuals to define difficulties in attention and memory. Some of these (e.g., the Conners) factor in sex into their age standardisation, which may have masked sex differences across the ICP and CCP groups in the current study.

Finally, while we focussed specifically on mental health as a transdiagnostic risk factor for poor cognitive function we relied on a single measure covering a limited set of symptoms, and did not measure other factors that impact on cognitive function, such as sleep. An important avenue for future research will be to conduct longitudinal studies with a wider range of measures to test the predictions of our functional account.

### Conclusion

The present study provides a novel exploration of the prevalence of ICPs in a sample of young people referred for learning difficulties based on practitioner referrals. Our findings reveal that almost half of all children referred for cognitive-related learning problems have an inconsistent profile of difficulties characterised by functional cognitive problems but preserved performance on cognitive tasks. Internalising and externalising problems were associated with these inconsistencies. Based on these findings, we propose that subjective reports of cognitive difficulties occurring in the absence of any performance deficits might arise, in part, as a functional problem developing from mental health problems. Future research into this account could expand our understanding of the functional pathways driving cognitive difficulties in struggling learners and provide a new outlook for clinical and educational assessment and interventions.

## Supplementary Information

Below is the link to the electronic supplementary material.

Supplementary file1 (DOCX 2486 KB)

## References

[CR1] Achenbach TM, Edelbrock CS (1981). Behavioral problems and competencies reported by parents of normal and disturbed children aged four through sixteen. Monographs of the Society for Research in Child Development.

[CR2] Albert MS, DeKosky ST, Dickson D, Dubois B, Feldman HH, Fox NC, Gamst A, Holtzman DM, Jagust WJ, Petersen RC, Snyder PJ, Carrillo MC, Thies B, Phelps CH (2011). The diagnosis of mild cognitive impairment due to Alzheimer’s disease: Recommendations from the National Institute on Aging-Alzheimer’s Association workgroups on diagnostic guidelines for Alzheimer’s disease. Alzheimer’s & Dementia: THe Journal of the Alzheimer’s Association.

[CR3] Alloway TP (2007). Automated working memory assessment.

[CR4] Alloway TP, Alloway RG (2010). Investigating the predictive roles of working memory and IQ in academic attainment. Journal of Experimental Child Psychology.

[CR5] American Psychiatric Association (2013). Diagnostic and statistical manual of mental disorders.

[CR6] Anderson KG (1997). Gender bias and special education referrals. Annals of Dyslexia.

[CR7] Astle DE, Bathelt J, Holmes J, CALM team (2019). Remapping the cognitive and neural profiles of children who struggle at school. Developmental Science.

[CR8] Barkley RA (1997). ADHD and the nature of self-control.

[CR9] Barkley RA (1999). Response inhibition in attention-deficit hyperactivity disorder. Mental Retardation and Developmental Disabilities Research Reviews.

[CR10] Bondi MW, Edmonds EC, Jak AJ, Clark LR, Delano-Wood L, McDonald CR, Nation DA, Libon DJ, Au R, Galasko D, Salmon DP (2014). Neuropsychological criteria for mild cognitive impairment improves diagnostic precision, biomarker associations, and progression rates. Journal of Alzheimer’s Disease: JAD.

[CR11] Brocki KC, Bohlin G (2006). Developmental change in the relation between executive functions and symptoms of ADHD and co-occurring behaviour problems. Infant and Child Development: An International Journal of Research and Practice.

[CR12] Brocki KC, Randall KD, Bohlin G, Kerns KA (2008). Working memory in school-aged children with attention-deficit/hyperactivity disorder combined type: Are deficits modality specific and are they independent of impaired inhibitory control?. Journal of Clinical and Experimental Neuropsychology.

[CR13] Bryant A, Guy J, Holmes J, the CALM Team (2020). Behavioural problems and mental ill-health in children struggling at school. Frontiers in Psychology, Developmental Psychology.

[CR14] Buckley R, Saling MM, Ames D, Rowe CC, Lautenschlager NT, Macaulay SL, Ellis KA, Australian Imaging Biomarkers and Lifestyle Study of Aging (AIBL) Research Group (2013). Factors affecting subjective memory complaints in the AIBL aging study: Biomarkers, memory, affect, and age. International Psychogeriatrics.

[CR15] Casey BJ, Durston S, Fossella JA (2001). Evidence for a mechanistic model of cognitive control. Clinical Neuroscience Research.

[CR16] Castellanos FX, Sonuga-Barke EJ, Milham MP, Tannock R (2006). Characterizing cognition in ADHD: Beyond executive dysfunction. Trends in Cognitive Sciences.

[CR17] Collins MW, Abeles N (1996). Subjective memory complaints and depression in the able elderly. Clinical Gerontologist.

[CR18] Conners CK (2008). Conners parent rating scale short form.

[CR19] Department for Education. (2018). *Special educational needs in England: January 2018*. https://assets.publishing.service.gov.uk/government/uploads/system/uploads/attachment_data/file/729208/SEN_2018_Text.pdf

[CR20] Dhuey E, Lipscomb S (2010). Disabled or young? Relative age and special education diagnoses in schools. Economics of Education Review.

[CR21] Dolcos F, Katsumi Y, Moore M, Berggren N, de Gelder B, Derakshan N, Hamm AO, Koster EHW, Ladouceur CD, Okon-Singer H, Pegna AJ, Richter T, Schweizer S, Van den Stock J, Ventura-Bort C, Weymar M, Dolcos S (2020). Neural 62 correlates of emotion-attention interactions: From perception, learning, and memory to social cognition, individual differences, and training interventions. Neuroscience and Biobehavioral Reviews.

[CR22] Donati G, Meaburn E, Dumontheil I (2021). Internalising and externalising in early adolescence predict later executive function, not the other way around: A cross-lagged panel analysis. Cognition & Emotion.

[CR23] Farina, F. R., Bennett, M., Griffith, J. W., & Lenaert, B. (2020). Fear of memory loss predicts increased memory failures and lower quality of life in older adults: preliminary findings from a fear-avoidance of memory loss (FAM) scale. *Aging & Mental Health*, 1–7. 10.1080/13607863.2020.1856780 33291990

[CR24] Fischer C, Schweizer TA, Atkins JH, Bozanovic R, Norris M, Herrmann N, Nisenbaum R, Rourke SB (2008). Neurocognitive profiles in older adults with and without major depression. International Journal of Geriatric Psychiatry.

[CR25] Follmer DJ (2018). Executive function and reading comprehension: A meta-analytic review. Educational Psychologist.

[CR26] Francis DA, Caruana N, Hudson JL, McArthur GM (2019). The association between poor reading and internalising problems: A systematic review and meta-analysis. Clinical Psychology Review.

[CR27] Fuhrmann, D., Simpson-Kent, I. L., Bathelt, J. (2020, January 10) CALM Team Holmes Joni Gathercole Susan Astle Duncan Manly Tom Kievit Rogier, Kievit RA. A hierarchical watershed model of fluid intelligence in childhood and adolescence. *Cerebral Cortex*, *30*(1), 339–352. 10.1093/cercor/bhz091 PMC7029679 31211362

[CR28] Gioia GA, Isquith PK, Guy SC, Kenworthy L (2000). Behaviour Rating Inventory of Executive Function - Ages 5–18 (BRIEF).

[CR29] Goodman A, Lamping DL, Ploubidis GB (2010). When to use broader internalising and externalising subscales instead of the hypothesised five subscales on the Strengths and Difficulties Questionnaire (SDQ): Data from British parents, teachers and children. Journal of Abnormal Child Psychology.

[CR30] Goodman, R. (1997). The strengths and difficulties questionnaire: a research note. *Journal of Child Psychology and Psychiatry*, *38*(5), 581–586. 10.1111/j.1469-7610.1997.tb01545.x 9255702

[CR31] Hänninen T, Reinikainen KJ, Helkala EL, Koivisto K, Mykkänen L, Laakso M, Pyörälä K, Riekkinen PJ (1994). Subjective memory complaints and personality traits in normal elderly subjects. Journal of the American Geriatrics Society.

[CR32] Harmer CJ, Clark L, Grayson L, Goodwin GM (2002). Sustained attention deficit in bipolar disorder is not a working memory impairment in disguise. Neuropsychologia.

[CR33] Hill NL, Mogle J, Wion R, Munoz E, DePasquale N, Yevchak AM, Parisi JM (2016). Subjective Cognitive Impairment and Affective Symptoms: A Systematic Review. The Gerontologist.

[CR34] Hiller RM, Young RL, Weber N (2014). Sex differences in autism spectrum disorder based on DSM-5 criteria: Evidence from clinician and teacher reporting. Journal of Abnormal Child Psychology.

[CR35] Holmes J (2012). Working memory and learning difficulties. Dyslexia Review.

[CR36] Holmes J, Bryant A, Gathercole SE, CALM Team (2019). Protocol for a transdiagnostic study of children with problems of attention, learning and memory (CALM). BMC Pediatrics.

[CR37] Holmes J, Guy J, Kievit R, Bryant A, Mareva S, Gathercole SE, The CALM Team (2020). Cognitive dimensions of learning in children with problems in attention, learning and memory. Journal of Educational Psychology.

[CR38] Holmes J, Hilton KA, Place M, Alloway TP, Elliott JG, Gathercole SE (2014). Children with low working memory and children with ADHD: Same or different?. Frontiers in Human Neuroscience.

[CR39] Holmes J, Mareva S, Bennett MP, Black MJ, Guy J (2021). Dimensions of Psychopathology in a Neurodevelopmental Transdiagnostic Sample..

[CR40] Hull L, Petrides KV, Mandy W (2020). The Female Autism Phenotype and Camouflaging: A Narrative Review. Review Journal of Autism and Developmental Disorders.

[CR41] Isquith PK, Roth RM, Gioia G (2013). Contribution of rating scales to the assessment of executive functions. Applied Neuropsychology: Child.

[CR42] Jeffries S, Everatt J (2004). Working memory: Its role in dyslexia and other specific learning difficulties. Dyslexia.

[CR43] Jenkins A, Tree JJ, Thornton IM, Tales A (2019). Subjective Cognitive Impairment in 55–65-Year-Old Adults Is Associated with Negative Affective Symptoms, Neuroticism, and Poor Quality of Life. Journal of Alzheimer's Disease: JAD.

[CR44] Jessen F, Amariglio RE, Buckley RF, van der Flier WM, Han Y, Molinuevo JL, Rabin L, Rentz DM, Rodriguez-Gomez O, Saykin AJ, Sikkes SAM, Smart CM, Wolfsgruber S, Wagner M (2020). The characterisation of subjective cognitive decline. The Lancet Neurology.

[CR45] Karalunas SL, Gustafsson HC, Fair D, Musser ED, Nigg JT (2019). Do we need an irritable subtype of ADHD? Replication and extension of a promising temperament profile approach to ADHD subtyping. Psychological Assessment.

[CR46] Landerl K, Kölle C (2009). Typical and atypical development of basic numerical skills in elementary school. Journal of Experimental Child Psychology.

[CR47] LeMoult J, Gotlib IH (2019). Depression: A cognitive perspective. Clinical Psychology Review.

[CR48] Lenaert, B., Bennett, M., Boddez, Y., & van Heugten, C. (2021). The influence of nocebo information on fatigue and urge to stop: An experimental investigation. *The Journal of Behavior Therapy and Experimental Psychiatry, (72).* 10.1016/j.jbtep.2021.101656 33839619

[CR49] Llewellyn DJ, Lang IA, Langa KM, Huppert FA (2008). Cognitive function and psychological well-being: Findings from a population-based cohort. Age and Ageing.

[CR50] Loe IM, Feldman HM (2007). Academic and educational outcomes of children with ADHD. Journal of Pediatric Psychology.

[CR51] Lui M, Tannock R (2007). Working memory and inattentive behaviour in a community sample of children. Behavioral and Brain Functions.

[CR52] Matthews K, Coghill D, Rhodes S (2008). Neuropsychological functioning in depressed adolescent girls. Journal of Affective Disorders.

[CR53] Manly T, Anderson V, Crawford J, George M, Underbjerg M, Robertson IH (2016). TEA-Ch 2: Test of Everyday Attention for Children.

[CR54] Martinussen R, Hayden J, Hogg-Johnson S, Tannock R (2005). A meta-analysis of working memory impairments in children with attention-deficit/hyperactivity disorder. Journal of the American Academy of Child & Adolescent Psychiatry.

[CR55] McWhirter L, Ritchie C, Stone J, Carson A (2019). Functional cognitive disorders: A systematic review. The Lancet. Psychiatry.

[CR56] Meltzer H, Gatward R, Goodman R, Ford F (2000). Mental health of children and adolescents in Great Britain.

[CR57] Minett TS, Da Silva RV, Ortiz KZ, Bertolucci PH (2008). Subjective memory complaints in an elderly sample: A cross-sectional study. International Journal of Geriatric Psychiatry.

[CR58] National Center for Education Statistics. (2019). *The Condition of Education 2019*. https://nces.ed.gov/pubs2019/2019144.pdf

[CR59] Nigg JT, Karalunas SL, Feczko E, Fair DA (2020). Toward a Revised Nosology for Attention-Deficit/Hyperactivity Disorder Heterogeneity. Biological Psychiatry. Cognitive Neuroscience and Neuroimaging.

[CR60] Nigg JT, Karalunas SL, Gustafsson HC, Bhatt P, Ryabinin P, Mooney MA, Faraone SV, Fair DA, Wilmot B (2020). Evaluating chronic emotional dysregulation and irritability in relation to ADHD and depression genetic risk in children with ADHD. Journal of Child Psychology and Psychiatry, and Allied Disciplines.

[CR61] Nigg JT, Sibley MH, Thapar A, Karalunas SL (2020). Development of ADHD: Etiology, Heterogeneity and Early Life Course. Annual Review of Developmental Psychology..

[CR62] Nigg JT, Willcutt EG, Doyle AE, Sonuga-Barke EJ (2005). Causal heterogeneity in attention-deficit/ hyperactivity disorder: Do we need neuropsychologically impaired subtypes?. Biological Psychiatry.

[CR63] Oberauer, K. (2019). Working memory and attention–A conceptual analysis and review. *Journal of cognition*, *2*(1). 10.5334/joc.58 PMC6688548 31517246

[CR64] Peng P, Fuchs D (2016). A meta-analysis of working memory deficits in children with learning difficulties: Is there a difference between verbal domain and numerical domain?. Journal of Learning Disabilities.

[CR65] Peng P, Namkung J, Barnes M, Sun C (2016). A meta-analysis of mathematics and working memory: Moderating effects of working memory domain, type of mathematics skill, and sample characteristics. Journal of Educational Psychology.

[CR66] Peng P, Wang C, Namkung J (2018). Understanding the Cognition Related to Mathematics Difficulties: A Meta-Analysis on the Cognitive Deficit Profiles and the Bottleneck Theory. Review of Educational Research.

[CR67] Reid JB, Kavanagh K, Baldwin DV (1987). Abusive parents' perceptions of child problem behaviors: An example of parental bias. Journal of Abnormal Child Psychology.

[CR68] Rogers M, Hwang H, Toplak M, Weiss M, Tannock R (2011). Inattention, working memory, and academic achievement in adolescents referred for attention deficit/hyperactivity disorder (ADHD). Child Neuropsychology.

[CR69] Rubia K (2018). Cognitive neuroscience of attention deficit hyperactivity disorder (ADHD) and its clinical translation. Frontiers in Human Neuroscience.

[CR70] Scheibe S, Blanchard-Fields F (2009). Effects of regulating emotions on cognitive performance: What is costly for young adults is not so costly for older adults. Psychology and Aging.

[CR71] Scheres A, Oosterlaan J, Geurts H, Morein-Zamir S, Meiran N, Schut H, Vlasveld L, Sergeant JA (2004). Executive functioning in boys with ADHD: Primarily an inhibition deficit?. Archives of Clinical Neuropsychology: THe Official Journal of the National Academy of Neuropsychologists.

[CR72] Schweizer S, Kievit RA, Emery T, Henson RN, Cam-CAN (2018). Symptoms of depression in a large healthy population cohort are related to subjective memory complaints and memory performance in negative contexts. Psychological Medicine.

[CR73] Sciutto MJ, Nolfi CJ, Bluhm C (2004). Effects of Child Gender and Symptom Type on Referrals for ADHD by Elementary School Teachers. Journal of Emotional and Behavioral Disorders.

[CR74] Sjöwall D, Roth L, Lindqvist S, Thorell LB (2013). Multiple deficits in ADHD: Executive dysfunction, delay aversion, reaction time variability, and emotional deficits. Journal of Child Psychology and Psychiatry..

[CR75] Slattery, E. J., Ryan, P., Fortune, D. G., & McAvinue, L. P. (2022). Unique and overlapping contributions of sustained attention and working memory to parent and teacher ratings of inattentive behavior. *Child Neuropsychology*, 1–23. 10.1080/09297049.2021.2022112 35000571

[CR76] Solanto MV, Abikoff H, Sonuga-Barke E, Schachar R, Logan GD, Wigal T, Hechtman L, Hinshaw S, Turkel E (2001). The ecological validity of delay aversion and response inhibition as measures of impulsivity in AD/HD: A supplement to the NIMH Multimodal Treatment Study of AD/HD. Journal of Abnormal Child Psychology.

[CR77] Sonuga-Barke EJS (2005). Causal models of attention-deficit/hyperactivity disorder: From common simple deficits to multiple developmental pathways. Biological Psychiatry.

[CR78] Stawski RS, Sliwinski MJ, Smyth JM (2006). Stress-related cognitive interference predicts cognitive function in old age. Psychology and Aging.

[CR79] Stone SL, Speltz ML, Collett B, Werler MM (2013). Socioeconomic Factors in Relation to Discrepancy in Parent versus Teacher Ratings of Child Behavior. Journal of Psychopathology and Behavioral Assessment.

[CR80] Swanson HL, Sachse-Lee C (2001). Mathematical problem solving and working memory in children with learning disabilities: Both executive and phonological processes are important. Journal of Experimental Child Psychology.

[CR81] Toplak, M., West, R. F., & Stanovich, K. E. (2017). The assessment of executive functions in attention-deficit/hyperactivity disorder (ADHD): Performance-based measures versus ratings of behavior. In M. Hoskyn, & G. Iarocci (Eds.), *Executive functions in children’s everyday lives: A handbook for professionals in applied psychology.* Oxford University Press. 10.1093/acprof:oso/9780199980864.003.0011

[CR82] Vaidya CJ, You X, Mostofsky S, Pereira F, Berl MM, Kenworthy L (2020). Data-driven identification of subtypes of executive function across typical development, attention deficit hyperactivity disorder, and autism spectrum disorders. Journal of Child Psychology and Psychiatry.

[CR83] Vaillancourt T, Haltigan JD, Smith I, Zwaigenbaum L, Szatmari P, Fombonne E, Waddell C, Duku E, Mirenda P, Georgiades S, Bennett T, Volden J, Elsabbagh M, Roberts W, Bryson S (2017). Joint trajectories of internalizing and externalizing problems in preschool children with autism spectrum disorder. Development and Psychopathology.

[CR84] Willcutt EG, Doyle AE, Nigg JT, Faraone SV, Pennington BF (2005). Validity of the executive function theory of attention-deficit/hyperactivity disorder: A meta- analytic review. Biological Psychiatry.

[CR85] Yeniad N, Malda M, Mesman J, van IJzendoorn MH, Pieper S (2013). Shifting ability predicts math and reading performance in children: A meta-analytical study. Learning and Individual Differences.

[CR86] Yoshimasu K, Barbaresi WJ, Colligan RC, Voigt RG, Killian JM, Weaver AL, Katusic SK (2012). Child- hood ADHD is strongly associated with a broad range of psychiatric disorders during adolescence: A population- based birth cohort study. Journal of Child Psychology and Psychiatry.

