# Supplementary material for: Inconsistencies between Subjective Reports of Cognitive Difficulties and Performance on Cognitive Tests are Associated with Elevated Internalising and Externalising Symptoms in Children with Learning-related Problems
Source: Res Child Adolesc Psychopathol. 2022 Jul 15;50(12):1557–72. doi: 10.1007/s10802-022-00930-4 (PMC9653343; doi:10.1007/s10802-022-00930-4)
Supplement: Supplementary file 1 — Supplementary file1 (DOCX 2486 KB) [file 10802_2022_930_MOESM1_ESM.docx]

**SUPPLEMENTAL MATERIAL**

**Figure S1**


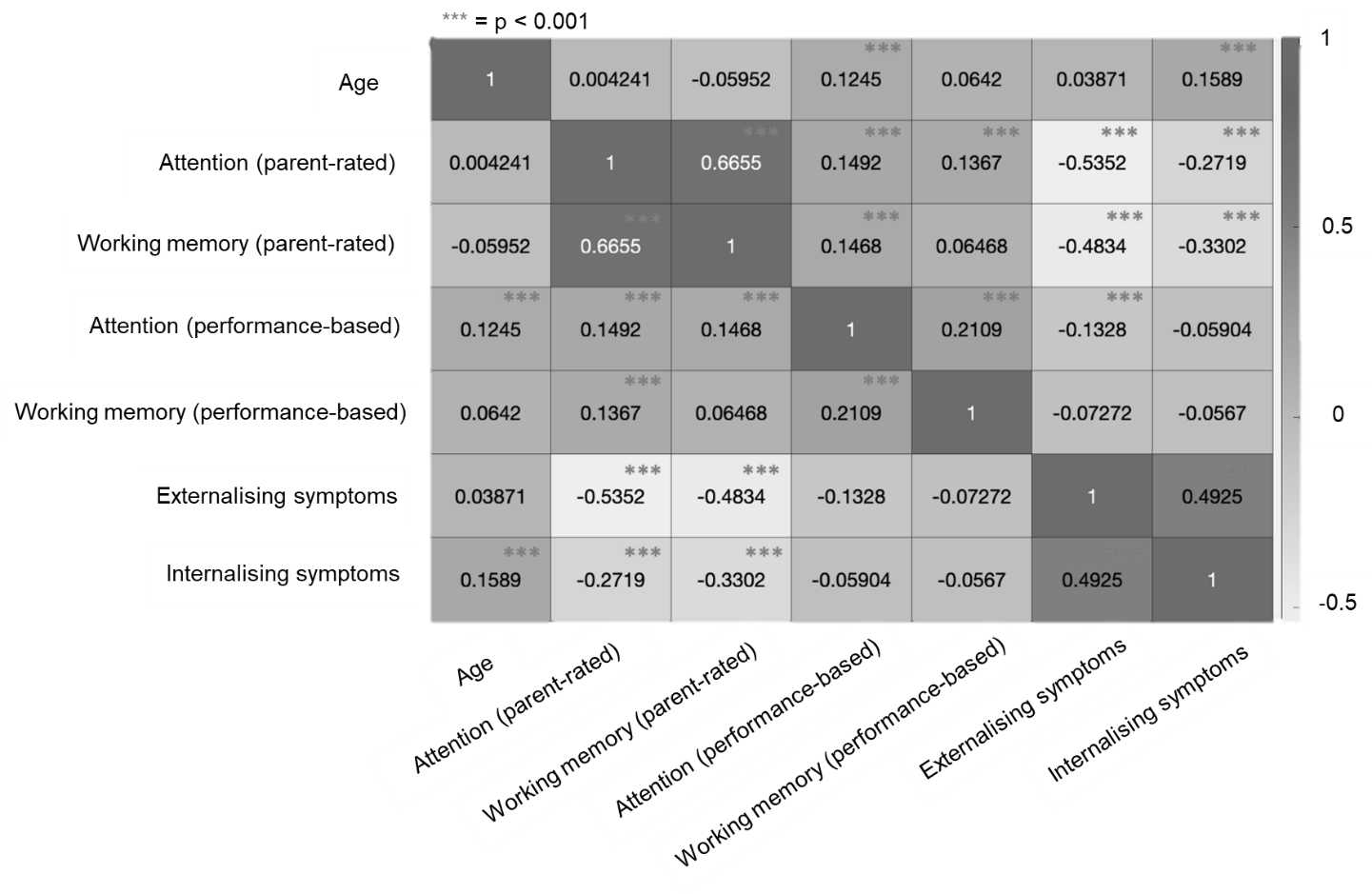

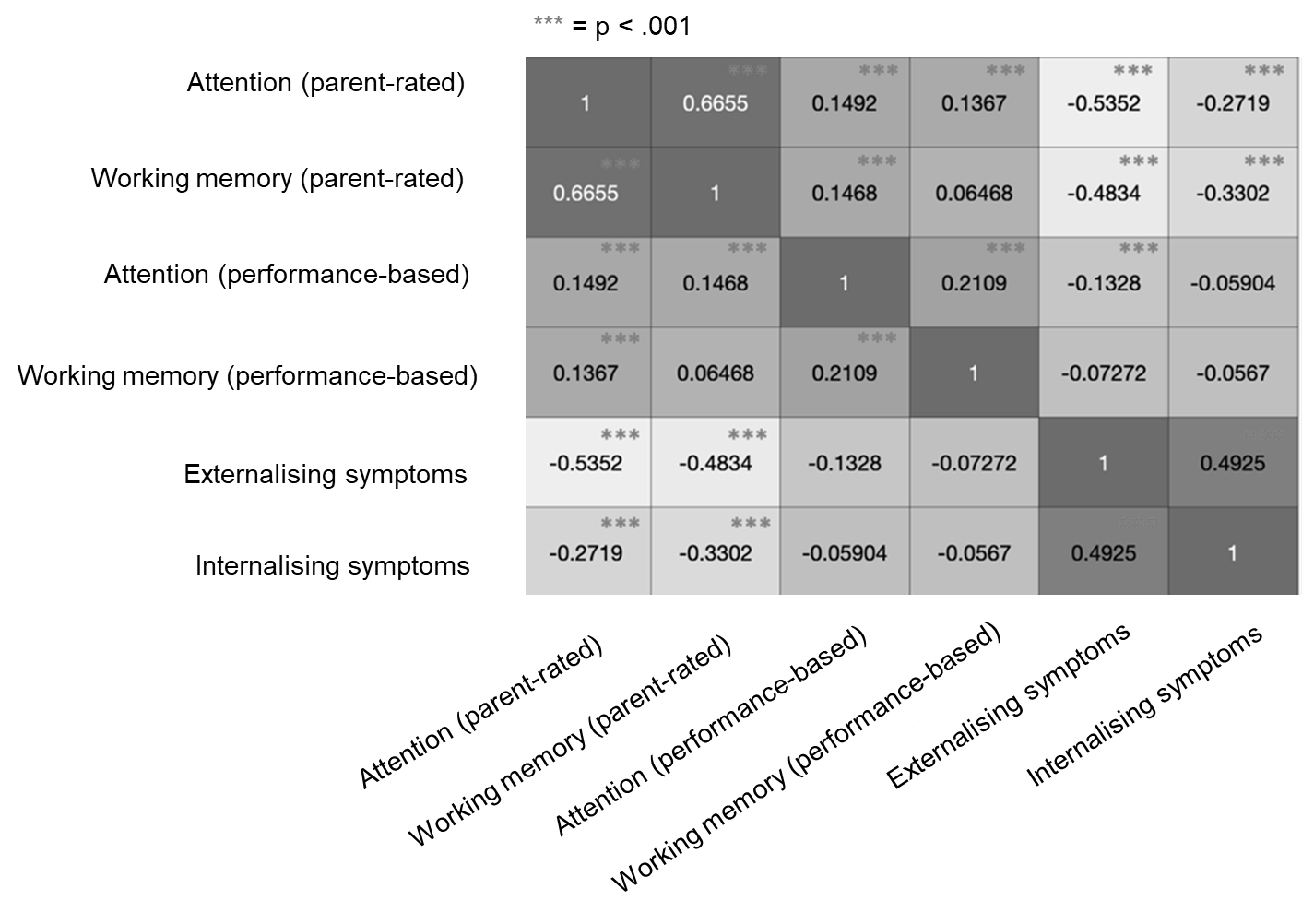

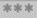


**Fig S1**: Correlations of all parent-rated cognitive measures, performance-based cognitive tasks and mental health measures used in the study. Red asterisks show significant associations, and darker shades represent a stronger positive association between two measures. Parent-rated measures have been reverse coded in the direction of performance-based measures, where low scores represent a greater cognitive impairment.

**Table S1.** Summary of psychometric information available for the performance-based measures of working memory and attention

|  | Performance-based measures | | | |
| --- | --- | --- | --- | --- |
|  | Barking sustained attention | Vigil sustained attention | Backward digit recall | |
| **Standardisation sample** |  |  |  |  |
| N | 394 | 621 | 746 | 351 |
| Age range (years) | 5 to 7 | 8 to 16 | 4 to 11 | 12 to 18 |
|  |  |  |  |  |
| **Scoring approach** | Scaled scores | Scaled scores | Standard scores | |
| Mean (SD) | 10 (3) | 10 (3) | 100 (15) | |
|  |  |  |  |  |
| **Reliability** |  |  |  |  |
| Internal consistency (range) | Between .50 and .80 | Between .50 and .90 | Between .60 and .80 | |
| Test-retest stability coefficient | Between .30 and .70 | Between .40 and .80 | .86 | |

Note. The Barking and Vigil sustained attention task was part of the automated Test of Everyday Attention in Children 2 (TEA-Ch2). The Backward Digit Recall task was part of the Automated Working Memory Assessment (AWMA). The data comprises the available psychometric information available in the test manuals for the standardisation of raw scores by the computer programmes running the performance-based tasks.

**Table S2.** Frequency distribution of participants who appear in each cognitive profile group for measures of working memory and attention

|  |  | Working Memory | | |
| --- | --- | --- | --- | --- |
|  |  | **CCP** | **ICP** | **Comparison** |
|  |  | n (%) | n (%) | n (%) |
| Inattention | **CCP** | 137 (19) | 171 (24) | 26 (4) |
|  | **ICP** | 74 (10) | 197 (28) | 27 (4) |
|  | **Comparison** | 0 (0) | 8 (1) | 23 (3) |

Note. Percentages are calculated from the whole sample with complete data (N = 715).

**Additional categorical analysis**

Internalising and externalising difficulties were also explored for inconsistent and consistent cognitive profiles (ICPs/CCPs) for the categorical approach using a 1.5 SD cut-off value to define performance-based cognitive difficulties for both attention and working memory (WM). This is reported below.

**Attention**

Participants with an ICP (M (SD) age = 116.16 (29.94) months) were older than those with a CCP (M (SD) age = 111.16 (29.72) months), t(642) = 2.00, p = .046, d = 0.17. A three-way ANOVA revealed a main effect of group (CCP vs. ICP vs. comparison group) on externalising symptoms (F(2,696) = 73.37, p < .001, partial eta^2^ =.174). Post-hoc t-tests indicated that the comparison group showed fewer externalising symptoms than both the CCP (t = 11.75, p < .001, d = 1.80) and ICP groups (t =11.34, p < .001, d = 1.59). However, there were no differences between the CCP and ICP groups, t = 2.05, p = .121. A three-way ANOVA revealed a main effect of group (CCP vs. ICP vs. comparison group) on internalising symptoms (F(2,696) = 19.68, p < .001, partial eta^2^ =.05). Post-hoc t-tests indicated that the comparison group showed fewer internalising symptoms than both the CCP (t = 5.58, p < .001, d = .85) and ICP groups (t =6.22, p < .001, d = .70). However, there were no differences between the CCP and ICP groups, t = 0.38, p = .99.

**Working memory**

Age did not differ across CCPs and ICPs, t(596) = .78, p = .44. A three-way ANOVA revealed a main effect of group (CCP vs. ICP vs. comparison group) on externalising symptoms (F(2,706) = 69.47, p < .001, partial eta^2^ =.16). Post-hoc t-tests indicated that the comparison group showed fewer externalising symptoms than both the CCP (t = 8.89, p < .001, d = 1.48) and ICP groups (t =11.30, p < .001, d = 1.15). However, there were no differences between the CCP and ICP groups, t = 1.71, p = .26. A three-way ANOVA revealed a main effect of group (CCP vs. ICP vs. comparison group) on internalising symptoms (F(2,706) = 18.84, p < .001, partial eta^2^ =.05). Post-hoc t-tests indicated that the comparison group showed fewer internalising symptoms than both the CCP (t = 4.14, p < .001, d = .68) and ICP groups (t =6.04, p < .001, d = .62). However, there were no differences between the CCP and ICP groups, t = 019, p = .99.
